# A deep learning based automatic two-dimensional digital templating model for total knee arthroplasty

**DOI:** 10.1186/s43019-024-00240-7

**Published:** 2024-11-27

**Authors:** Jaeseok Park, Sung Eun Kim, Back Kim, Sanggyu Lee, Jae-Jun Lee, Du Hyun Ro

**Affiliations:** 1 College of Medicine, Seoul National University, Seoul, South Korea; 2 Department of Orthopaedic Surgery, Seoul National University College of Medicine, Seoul, South Korea; 3 Department of Orthopaedic Surgery, Seoul National University Hospital, Seoul, South Korea; 4 Department of Computer Science and Engineering, Seoul National University, Seoul, South Korea; 5 CONNECTEVE Co., Ltd, Seoul, South Korea; 6 Innovative Medical Technology Research Institute, Seoul National University Hospital, Seoul, South Korea

**Keywords:** Total knee arthroplasty, Artificial intelligence, Preoperative templating, Implant size prediction

## Abstract

**Background:**

Preoperative templating is an important step for total knee arthroplasty (TKA), facilitating hospital personnel in the anticipation and preparation of necessary surgical resources. Despite its importance, this process currently lacks automation. This study aimed to develop an artificial intelligence (AI) model to automate implant size prediction.

**Methods:**

A total of 13,281 (2938 anteroposterior, 10,343 lateral) knee radiographs obtained from the authors’ institute were utilized for model training, with 2302 (1034 anteroposterior, 1268 lateral) images set apart for validation and testing. The templating AI model integrates a pipeline composed of multiple steps for automated implant size estimation. To predict implant size, anterioposterior (AP) and lateral radiograph predictions were merged, selecting the smaller of the predicted sizes to prevent implant overhang. The model’s size predictions were validated with 81 real TKA data set apart from the training data, and its accuracy was compared to that of manual templating by an orthopedic specialist. Predictions matching the actual implanted sizes were labeled “exact” and those within one size, “accurate.” The influence of patient characteristics on the model’s prediction accuracy was also analyzed. The measurement time elapsed for implant sizing was recorded for both the AI model and the orthopedic specialist. Implant position predicted by the model was validated by comparing insert locations with postoperative images.

**Results:**

Compared with data from 81 actual TKA procedures, the model provided exact predictions for 39.5% of femoral and 43.2% of tibial components. Allowing a one-size margin of error, 88.9% of predictions were deemed “accurate” for both components. Interobserver reliability (Cohen’s kappa) were 0.60 and 0.70 for femoral and tibial implants, respectively, both classified as “substantial.” The orthopedic specialist produced results accurate within one-size margin of error in 95.1% and 100% of cases for femoral and tibial components, respectively. Interobserver reliability between the orthopedic specialist and ground truth was 0.76 and 0.8 for femoral and tibial components, respectively. The measurement time per case was 48.7 s for the AI model, compared with 97.5 s for the orthopedic specialist. Compared with postoperative radiographs, predicted implant position had an error of less than 4 mm on average.

**Conclusions:**

An AI-based templating tool for TKA was successfully developed, demonstrating satisfactory accuracy and efficiency. Its application could significantly reduce the clinical workload in TKA preparation.

## Introduction

Surgical planning and templating prior to total knee arthroplasty (TKA) are important steps for simulating bony cuts, implant positioning, and selecting appropriate implant sizes. These steps not only streamline the surgical procedure but also ensure the availability of suitable implants and prevent unexpected complications [1–3]. Two-dimensional (2D) digital templating, using plain radiographs, stands as the most widely used method for determining the mechanical axis of the knee and predicting implant locations and component sizes [2, 4]. This method allows surgeons to double-check their surgical plans with templated data, promoting accurate implant positioning and sizing, which in turn contributes to the implant’s longevity and reduces the risk of adverse outcomes, such as femoral notching—a known cause of periprosthetic fractures [4]. Additionally, it reduces postoperative pain and complications and reduces the number of required surgical trays, cutting down on expenses and the space needed for implant preparation [5, 6].

Despite its benefits, digital templating is time-consuming and may not always provide precise estimations, leading to mismatches with the actual implant size determined during surgery [7]. Previous research has highlighted these inefficiencies caused by having to fetch additional implants during surgery, which lead to increased cost, preparation time, and operating time [8].

To address these issues, three-dimensional (3D) templating using computed tomography (CT) images has been proposed and yielded promising results, with an exact implant size match rate of 80% [9]. However, routine preoperative CT scanning for every TKA patient raises concerns regarding time, cost, and radiation exposure.

In response to these challenges, efforts have been made to automate the templating process to improve efficiency and accuracy. Although an automated 2D templating algorithm has been developed, it demonstrated significantly lower accuracy compared with manual 2D templating [10]. A previous study suggested that automatic templating based on a 3D image reconstructed from simple X-rays could surpass the accuracy of the traditional manual 2D process [1]. However, this algorithm was developed on the basis of images of healthy knees, which may not be universally applicable to osteoarthritic knees. [11] Additionally, a 3D image may not provide the simplicity and intuitiveness of the 2D method commonly in use.

Therefore, this study aimed to develop an automated 2D TKA templating tool that visualizes the mechanical axis of the knee, implant positioning, and bone resection guides in a manner similar to manual 2D templating. We validated the model’s size predictions with actual implanted TKA sizes and compared its accuracy and elapsed time against manual templating performed by an experienced surgeon, hypothesizing that the tool’s accuracy would be comparable to the accuracy of manual templating while saving time.

## Materials and methods

This study received approval from the institutional review board (IRB) of the authors’ institute (IRB No. 2405-168-1540). Informed consent was waived due to the retrospective nature of the study. We conducted a retrospective analysis of patients who underwent primary TKA from December 2022 to November 2023. Inclusion criteria were those diagnosed with osteoarthritis and treated with the Triathlon system (cruciate retaining; Stryker, MI, USA), which provides femoral and tibial components ranging from sizes 1 to 8. Eligibility was limited to cases with all preoperative full-leg, AP and lateral knee X-rays that included a calibration ruler. The exclusion criteria applied to TKA cases marked by severe bony attrition or those requiring the use of blocks or stem augments. Minimum sample size necessary was calculated as 49 knees according to sample size calculation for unmatched case–control studies.

### Overview of the algorithm for automatic templating

The artificial intelligence (AI) based templating model used in this study comprises four distinct modules: calibration, knee joint line detection, landmark detection, and bone segmentation. These modules are designed to process both anteroposterior (AP) and lateral radiographic images. The calibration module recognizes objects of a predefined size to counteract the effect of object magnification, enabling precise estimation of the femur and tibia sizes. The knee joint line and landmark detection module recognizes the orientation of the knee joint and accordingly determines the direction and location of the implant. The bone segmentation module estimates the cortical contours of the femur and tibia, fine-tuning the implant’s position to ensure the cortical contour of the bone matches the articular surface of the implant accurately. When full leg AP and lateral X-rays are input into the AI model, it automatically determines implant size and location on each dimension. To prevent overhang, the lesser size from the 2 dimensions is chosen as the merged prediction. Additionally, a hovering implant image of the predicted size is superimposed on the original image, visualizing the model’s suggestions for location and alignment.

### Coronal plane templating

 In the coronal plane, the templating AI model begins by determining the medial and lateral borders of the implant, recommending a size on the basis of its mediolateral (ML) width. We adopted a convolutional neural network (CNN) model trained by Jo et al., which accurately identifies specific landmarks to calculate the mechanical axes of the femur and tibia [12]. The landmarks used in the study are the femoral head center and trochlear groove of femur to determine the mechanical axis of femur, and the medial and lateral edges of the tibial plateau and talar dome center for the mechanical axis of tibia.

The subsequent step involves a CNN-based segmentation model that generates bony masks of the femur and tibia from a knee AP radiograph. This model, based on the Swin Transformer with pretrained weights [13], was additionally trained on 2938 images, validated on 734 images, and tested on 300 images, allowing for the extraction of precise bone contours from the generated masks. An additional model was trained to determine the tibial joint line from a cropped image of the knee joint. It was trained on 2152 images, validated on 537 images, and tested on 572 images. All CNN-based models mentioned in this study used randomly split train/validation/test images.

**Fig. 1:**
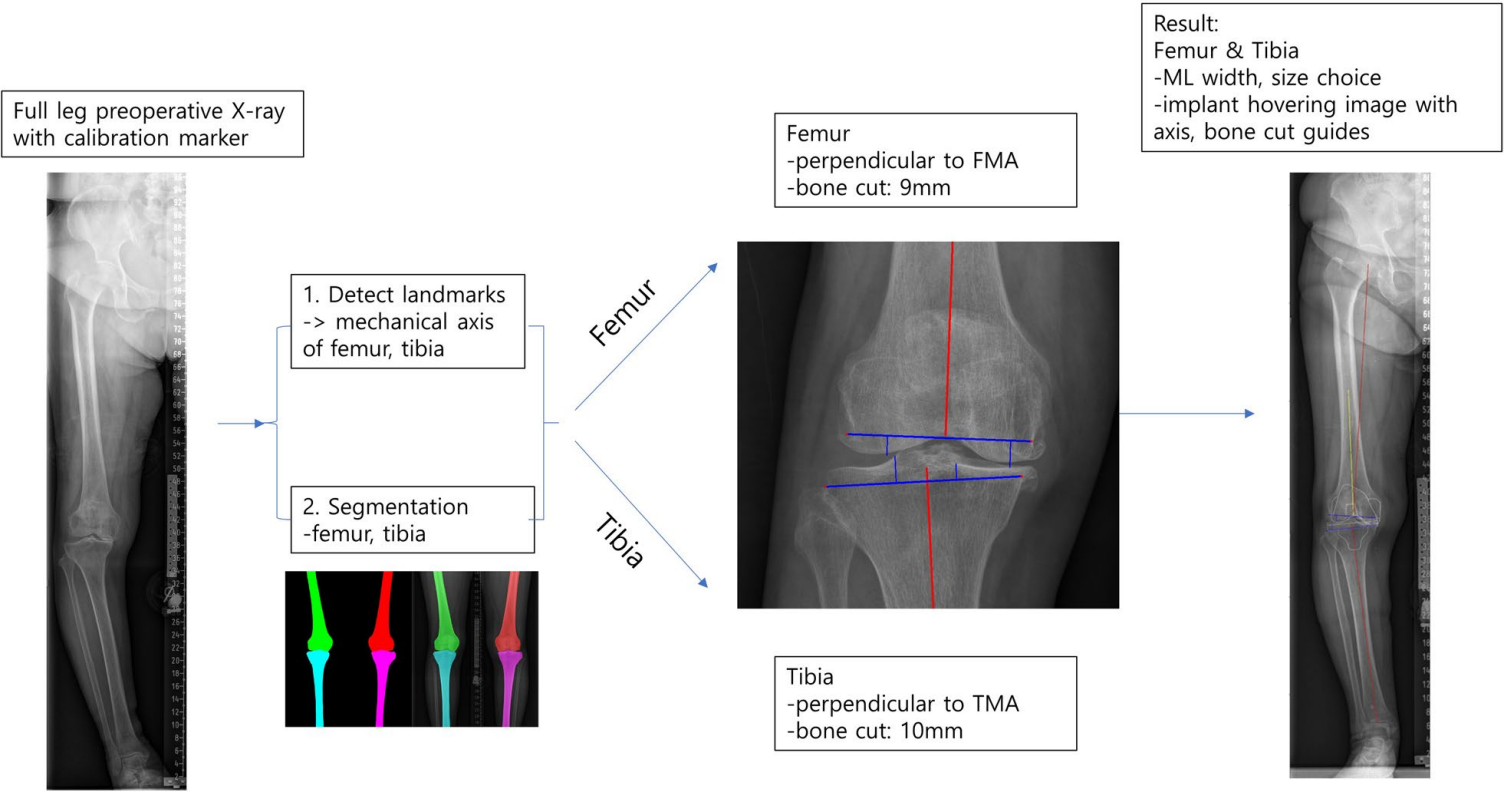
Pipeline of automatic templating on Coronal plane. *FMA* femur mechanical axis, *TMA* tibia mechanical axis, *ML* mediolateral

**Fig. 2:**
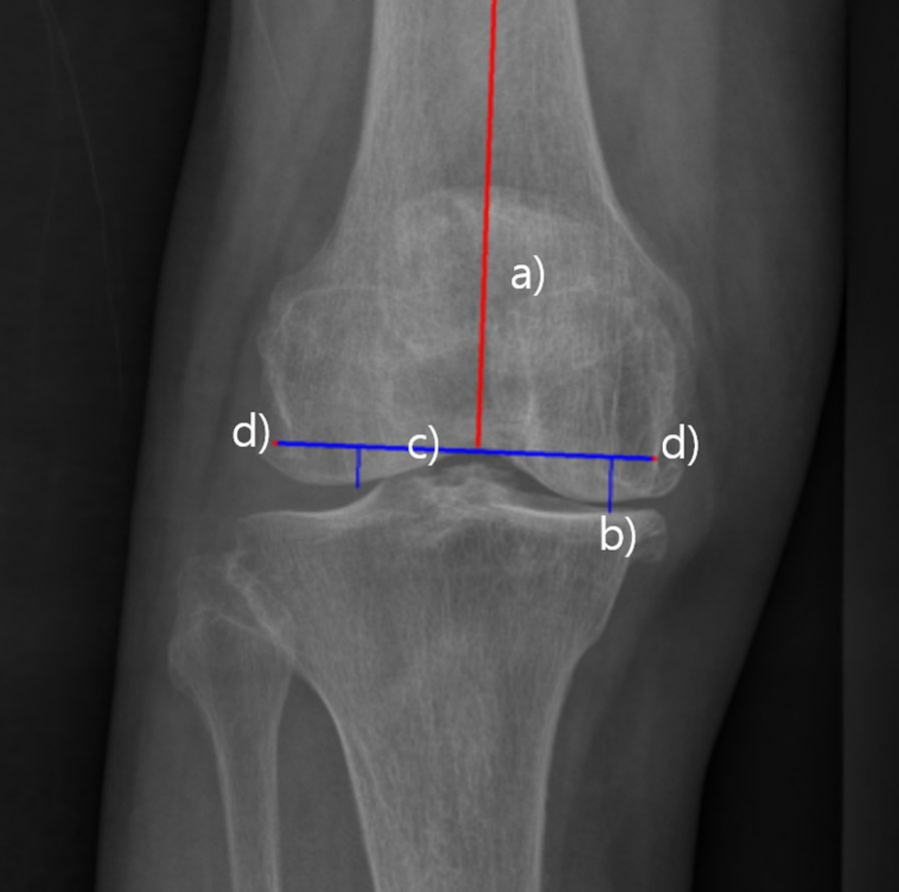
Calculations of femur width on Coronal plane. **a** FMA determined by landmarks (red) **b** Furthest point of femoral joint contour when projected to FMA **c** Upright line to axis at the point translated 9 mm upward from furthest point in (**b**). **d** Intersections of perpendicular line from (**c**) with bony contours are medial/lateral insert points

**Fig. 3:**
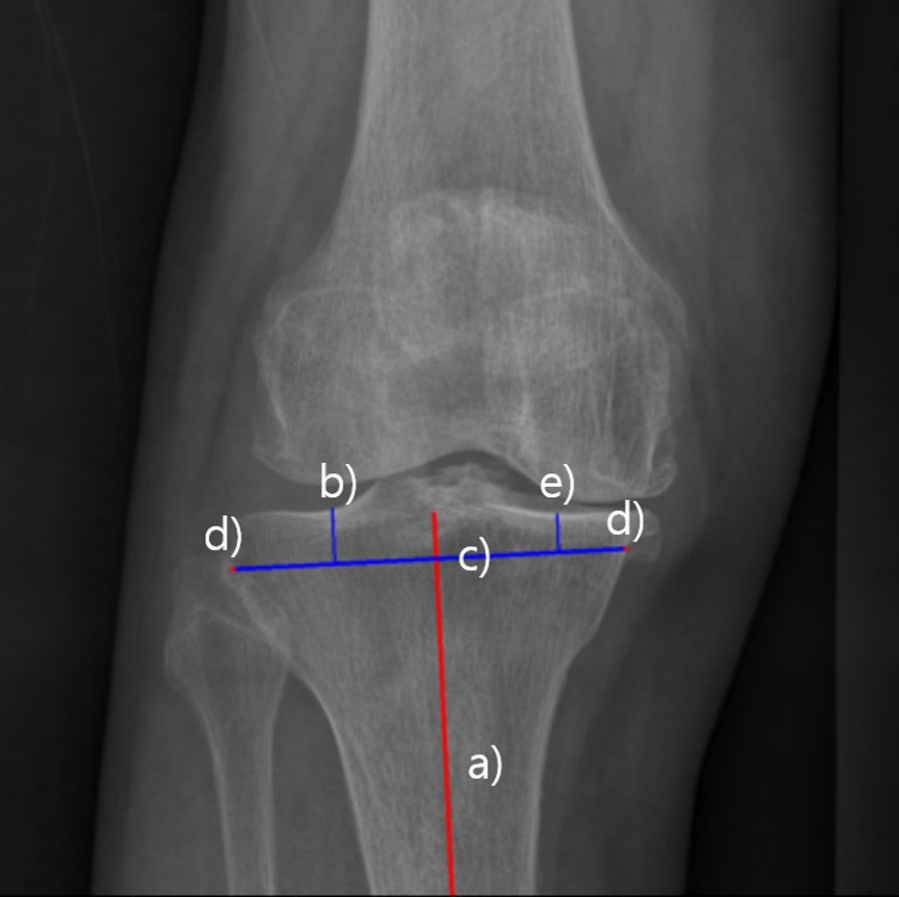
Calculations of tibia width on coronal plane. **a** TMA determined by landmarks (Red) **b** Furthest point of tibial joint contour when projected to TMA **c** Perpendicular line to axis at the point translated 10 mm downward from furthest point in (**b**). **d** Intersections of perpendicular line from (**c**) with bony contours are medial/lateral insert points. **e** Calculate resection depth on lesser side, if < 2 mm, and recalculate with MPTA = 88°

Using the identified mechanical axes and bone contours of the femur and tibia, an automatic calculation algorithm was developed. This algorithm, based on the concept of mechanical alignment [14], aims for a neutral hip-knee alignment (HKA). The algorithm prescribes a fixed bone resection amount of 9 mm in at least one compartment of the knee, corresponding to the implant’s thickness. In cases of bone attrition or severe varus knees, alignment of the tibial component is adjusted by 2° to secure a minimum bone resection of 2 mm on the lesser, in this case medial, side. The calculation process of coronal plane templating is summarized with example images in Figs. 1, 2 and 3.

### Sagittal plane templating

**Fig. 4:**
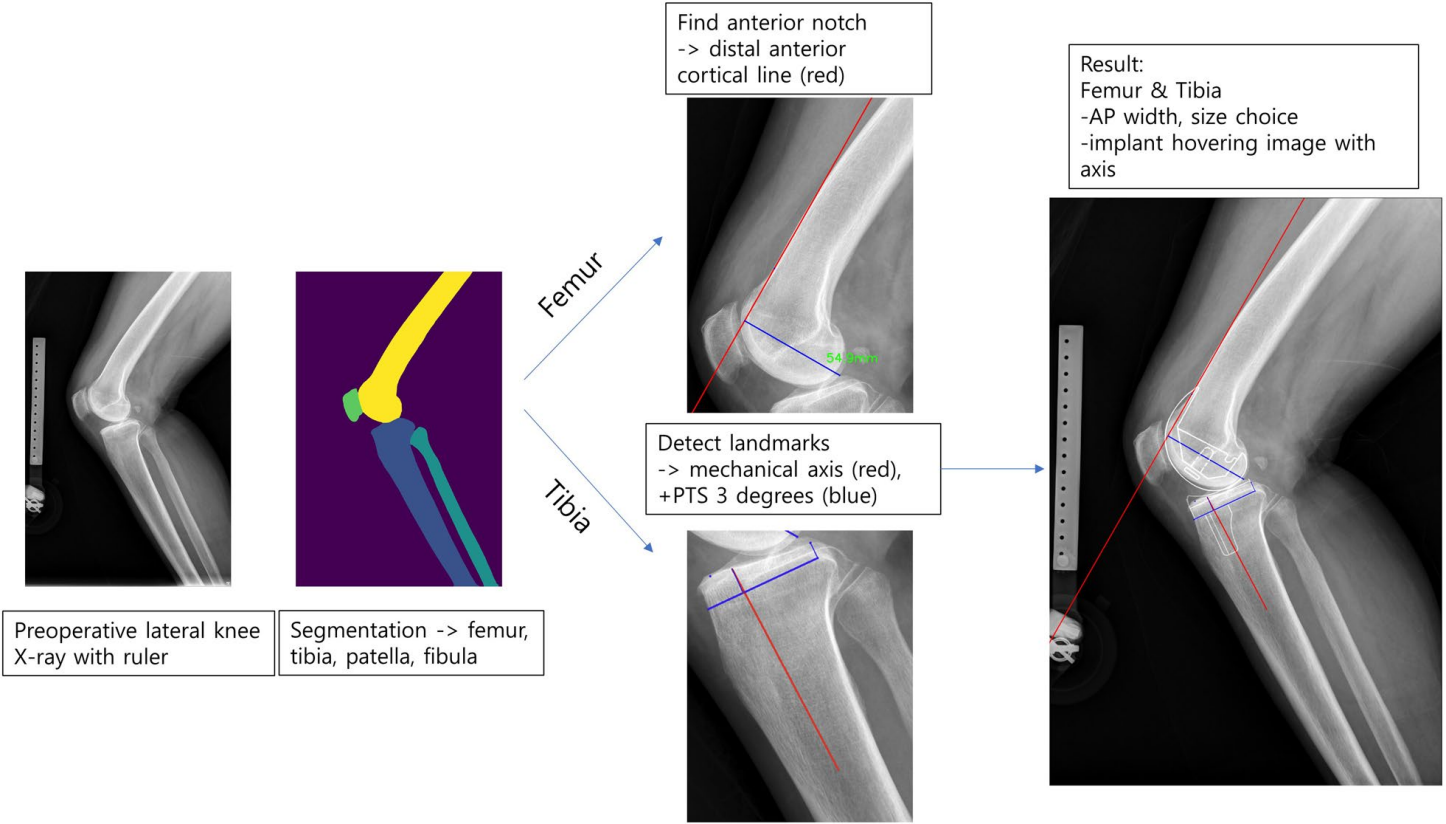
Pipeline of automatic templating on sagittal plane. *PTS* posterior tibial slope, *AP* anterioposterior

In this phase, the Templating AI model calculates the anterior and posterior borders of the implant using a calibrated lateral knee X-ray, thereby determining the size choice based on the implant’s anterior-posterior (AP) width. The workflow for this planning phase is detailed in Fig. 4.

Initially, a CNN-based segmentation model to generate bony masks of the femur, tibia, patella, and fibula was employed. This segmentation model, using the Swin Transformer architecture with pretrained weights as on the coronal plane, was further trained with 1066 images, validation on 261 images, and testing on 337 images.

For femoral component planning, the anterior cortical line of the distal femur was used for alignment. This approach to femoral component positioning is aimed at reducing the risk of femoral notching [4]. The AP length of the femur is determined by measuring the longest distance from the anterior cortical line to a point on the distal femoral condyle.

To size the tibial component, identification of the anterior and posterior points of the tibial joint surface and the tibial anatomical axis was necessary. A landmark identification model based on HRNet [15] was further trained on 9277 images, validated on 500 images, and tested on 170 images. The tibial component is then aligned to achieve a posterior tibial slope of 3° relative to the tibia anatomical axis, assuming a posterior-stabilized implant.

### Performance evaluation of size estimation

The accuracy of implant size estimations by the templating AI model was retrospectively evaluated by comparing them with 81 actually implanted TKAs, which served as the ground truth. A prediction was labeled as “exact” if the predicted and implanted components were of the same size. It was considered “accurate” if the prediction was within one size of the implanted size. Predictions not meeting these criteria were deemed “inaccurate” and further categorized as either overestimates or underestimates. Additionally, the total time required for templating AI to calculate implant size was measured for each knee.

For comparison, the same radiographs were evaluated by an orthopedic specialist with 5 years of experience in knee surgery. The surgeon performed 2D templating and estimated implant size, which were then compared to real surgical choices and classified into exact, accurate, and inaccurate as above. Time duration was also measured for comparison with templating AI.

The coronal plane, sagittal plane, and final predictions were each compared with the actual implanted size to investigate whether a single view could be more reliable for femoral or tibial size choice. Also, whether patient factors such as gender, age, and body mass index (BMI) significantly affected the accuracy of the AI model was analyzed. BMI was classified into underweight (< 18.5), healthy weight (18.5–22.9), overweight (23–24.9), obesity type 1 (25–29.9), and type 2 ($\ge$ 30) according to WHO Asian-Pacific cutoff points.

### Performance evaluation of implant positioning

The accuracy of implant positioning by templating AI was evaluated via comparison to post-operative radiographs. Knee AP and Lateral radiographs of the same 81 TKAs within were collected 2 weeks postoperatively, and implant edge points were marked manually for the femoral and tibial component on each radiograph. Then, postoperative radiographs were aligned and rescaled to match those of the preoperative radiographs. Rescaling and translation were done by manually marking corresponding points, while rotation was adjusted by aligning segmentation masks. These calculated transformations were applied to the postoperative image and implant coordinates to first verify proper overlapping with the preoperative images. Once overlapping was confirmed, the distance between the implant coordinates and model predictions were calculated. The midpoint of lateral and medial implant insertion points was used for comparison of AP femoral, AP tibial, and Lat tibial implant predictions. Predictions of femoral component on the sagittal plane were compared using the most posterior point of the implant from the anterior axis. Moreover, model predicted implant alignment was compared with postoperative alignment.

### Statistical analysis

The performance of the segmentation models was evaluated using the Intersection over Union (IoU) score and Dice coefficient for both the femur and tibia. Cohen’s kappa with quadratic weights was used to assess the inter-observer reliability of the model and orthopedic specialist’s size estimations, with in-surgery choices serving as ground truth. The values are interpreted as follows: below 0, poor reliability; 0.01–0.20, slight; 0.21–0.40, fair; 0.41–0.60, moderate; 0.61–0.80, substantial; and 0.80–1, almost perfect reliability [16]. The Chi-squared test and Student’s *t*-test was used to analyze the effect of categorical and continuous variables on model accuracy. The accuracy and time duration of the AI model and manual templating were each compared by McNemar’s test and Student’s *t*-test, respectively. Statistical significance for all statistical tests were $p<0.05$.

## Results

**Fig. 5:**
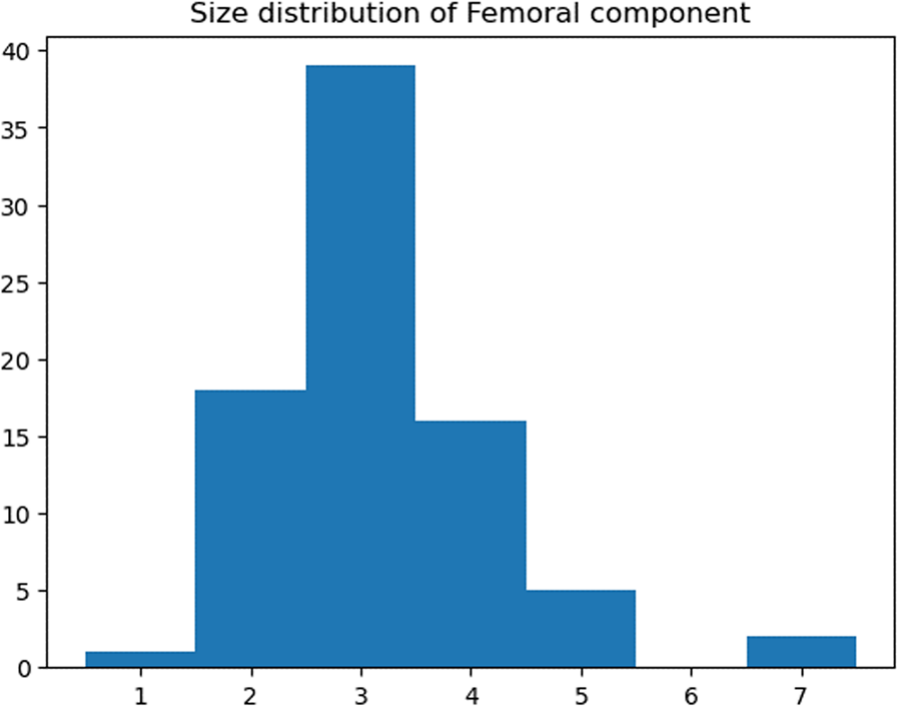
Size distribution of femoral component chosen in surgery

**Fig. 6:**
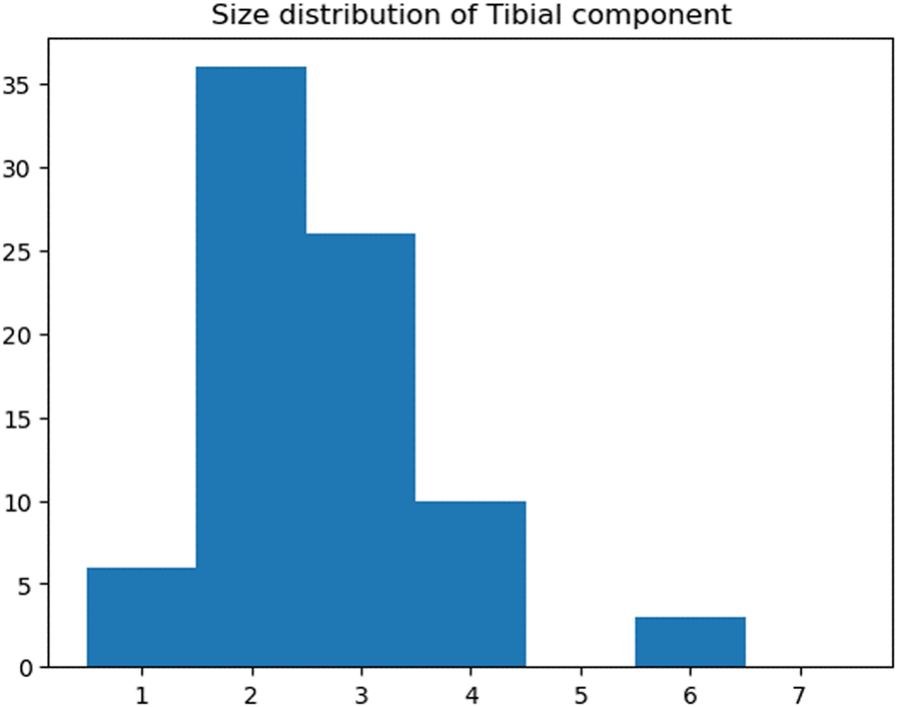
Size distribution of tibial component chosen in surgery

A total of 81 TKAs were performed on 72 patients that satisfied the inclusion criteria. Of these, nine patients received staged bilateral total knee arthroplasties. Patient characteristics are summarized in Table 1. The size distribution of femoral and tibial components of the 81 TKAs are shown in Figs. 5 and 6.

**Table 1:** Characteristics of patients

*N* = 72	Mean [standard deviation (SD)]
Age (years)	72.0 (7.6)
Sex	
Male (%)	12.5
Body mass index	26.16 (4.9)
Bilaterality (%)	
Bilateral	12.5
Unilateral	87.5%
Side	
Right (%)	54.3%
Alignment (%)	
Neutral	8.6
Varus (> 3°)	77.7
Valgus (< -3°)	13.6%

Comparison of AI model size predictions and actual size are presented in Tables 2 and 3. Exact match of size was observed in 39.5% and 43.2% of cases for the femur and tibia respectively. With one size of error accepted as “accurate,” accurate estimations were both 88.9% for the femur and tibia. Cohen’s kappa was 0.60 and 0.70 for the femur and tibia, each classified as “moderate” and “substantial”.

**Table 2:** Accuracy of model size predictions for femoral component

	Model (integrated)	Model (coronal plane)	Model (sagittal plane)
Exact	32 (39.5%)	19 (23.5%)	31 (38.3%)
Accurate	72 (88.9%)	63 (77.8%)	66 (81.5%)
Overestimates	2 (2.5%)	11 (13.6%)	15 (18.5%)
Underestimates	7 (8.6%)	7 (8.6%)	0

“Exact” refers to the cases where model predictions were equal to actual size. ‘Accurate’ predictions are when predictions are exact, or within one size margin of error

**Table 3:** Accuracy of model size predictions for tibial component

	Model (integrated)	Model (coronal plane)	Model (sagittal plane)
Exact	35 (43.2%)	22 (27.2%)	27 (33.3%)
Accurate	72 (88.9%)	62 (76.5%)	70 (86.4%)
Overestimates	1 (1.2%)	15 (18.5%)	7 (8.6%)
Underestimates	8 (9.9%)	4 (4.9%)	4 (4.9%)

The accuracy of isolated predictions from coronal and sagittal planes were both lower than that of the merged prediction, which was determined by the lesser of two. Coronal plane predictions were accurate in 77.8% and 76.5% accurate for the femur and tibia, while sagittal plane predictions were accurate in 81.5% and 86.4% of cases.

Manual templating (Table 4) was accurate within one size in 95.1% and 100% for the femur and tibia respectively, and an exact match was observed in 54.3% and 50.6%. Cohen’s kappa was 0.76 and 0.8 for the femur and tibia respectively, both classified as ‘substantial’. Compared with the AI model, accuracy within one size was significantly higher in manual templating for the tibial component (*p* < 0.01), but not the femoral component (*p* = 0.27). Exact match of size was not significantly higher in manual templating compared to the AI model for both the femur and tibia (*p* = 0.06, 0.39).

**Table 4:** Accuracy of manual 2D templating by surgeon

	Femur	Tibia
Exact	44 (54.3%)	41 (50.6%)
Accurate	77 (95.1%)	81 (100%)
Overestimates	1 (1.2%)	0
Underestimates	3 (3.7%)	0

The AI model produced results in 48.7 s per image on average with a standard deviation of 1.9 s. Manual templating was measured to take 97.5 s per image on average. The AI model had a significantly short time duration per image. (*p* < 0.01).

Sex, BMI class, and age had no effect on the automatic templating accuracy for the both femur and tibia (*p* > 0.05).

Model predictions of implant position differed by 3 ~ 4 mm on average compared with postoperative AP and Lat radiographs. Implant alignment had an error less than 2° on average in the coronal plane and for the tibia on sagittal plane, while femoral alignment on the sagittal plane had an average error of 5.1° (Table 5).

**Table 5:** Accuracy of implant positioning in AP and Lat radiographs

	Femur position (mm)	Femur alignment (°)	Tibia position (mm)	Tibia alignment (°)
AP radiographs	3.9 (2.2)	1.8 (1.7)	4.0 (2.5)	2.0 (1.7)
Lateral radiographs	3.6 (2.1)	5.1 (3.3)	3.5 (2.0)	1.6 (1.7)

*AP* anterioposterior, *Lat* lateral

## Discussion

The most important finding of this study is that the automated 2D templating model’s accuracy was slightly lower but almost comparable to the manual measurements of the orthopedic specialist. Our model demonstrated an accuracy (within 1 size) of 88.9% (72/81) for both the femur and tibia, lower than that of an experienced orthopedic surgeon. However, the difference was only significant for the tibial component. Moreover, as preoperative implant sizing is often changed in the surgical field, accuracy of the present AI model may be satisfactory for practical use.

Prior 2D automated templating approaches have shown an accuracy within ± 1 size of 51.2% and 71.2%, respectively, for the femur and tibia, both significantly lower than that of a surgeon (97.6% and 94.2%) [10]. The AI model developed in the present study has lessened the gap and has proved higher efficiency compared with manual templating. Other retrospective studies that have validated image-based TKA size estimation methods with surgical choices are summarized in Table6. In addition to these imaging-based approaches, studies with anthropometric approaches [17] have explored the use of multivariate linear regression and machine learning to predict implant component size without radiographs, relying solely on patient measurements, such as height and ankle volume. The model developed by Naylor et al. [18] reached an accuracy of 83% and 96% within ± 1 size range, but such approaches lack the ability to visualize mechanical axes and implant insert locations, therefore being less practical in the clinical setting.

**Table 6:** Reported accuracy of 2D and 3D templating for TKA

Author	Number of validation set	Method	Exact	Exact	Accurate	Accurate
			Femur %	Tibia %	Femur %	Tibia %
Seaver et al. [10]	125	Manual 2D templating	56	61.6	97.6	95.2
Riechelmann et al. [7]	482	Manual 2D templating	55.2	52.9	96.5	94
Seaver et al. [10]	125	Automatic 2D templating	19.2	26.4	51.2	71.2
Pietrzak et al. [19]	31	Manual 3D templating (CT)	96.6	93.1	100	100
Marchand et al. [9]	239	Manual 3D templating (CT)	80	78	100	100
Klag et al. [1]	202	Automated 2D–3D pipeline	38	93.1	84	99.5

*2D* two-dimensional, *3D* three-dimensional, *CT* computed tomography

Our model had a tendency to underestimate component size on average, as the final prediction was chosen as the lesser of coronal and sagittal predictions to prevent overhang. However, sagittal predictions on femoral component size and coronal predictions on tibial component size tended to be higher than actual size. Klag et al. [1] suggests that general overestimation of femoral component size may be owing to the difficulty in prediction of bone loss or soft tissue gaps in case of severe deformities. Additionally, sagittal alignment of the femoral component may vary depending on the operator and individual patient’s anatomy to prevent both notching and overstuffing. The tendency of sagittal predictions to overestimate femoral component size may result from its primary alignment goal, which was set as minimizing anterior notching.

Compared with manual templating, a significant decrease in time duration was observed. While it may be obvious, no previous study had compared an automated tool with manual templating in terms of elapsed time per processed image.

No patient demographic was found to affect model accuracy. Such irrelevance of age, sex, or BMI on templating accuracy is consistent with previous studies [7].

While most previous studies focus on the accuracy of implant size prediction, we have gone further to address the accuracy of implant positioning. We verified that our predictions of implant locations on both sagittal and coronal planes deviated from surgical choices by 3 ~ 4 mm on average. With minor modifications, model recommendations for the size and location could be of clinical value.

However, our study has limitations. Lack of precision in bone segmentation was pronounced when dealing with severe bony deformities. Osteophytes and irregular bone contours made it difficult to exactly determine bone contours. Additionally, our study only included patients with Triathlon Knee System implants who received surgery in a single institute, all of South Korean ethnicity. This may affect the generalizability of our results. A multicentered, multinational study would externally validate the model’s performance. Moreover, while the presently developed AI model deals with not only size estimation but also implant positioning, validation of position and alignment results was unaccomplished owing to the difficulties in setting ground truth. Such results may prove the clinical value of our automated 2D templating tool in the preparation of total knee arthroplasty.

## Conclusions

This study aimed to develop an automated 2D preoperative templating model for total knee arthroplasty and evaluate its accuracy in predicting implant size and location by comparison with actual surgery records. Allowing a one-size margin of error, an accuracy of 88.9% was achieved for implant sizing, with significantly reduced time elapsed for templating compared with manual templating.

## Data Availability

The datasets used and/or analyzed during the current study are available from the corresponding author upon reasonable request.

## Abbreviations

2D: Two-dimensional
3D: Three-dimensional
AI: Artificial intelligence
AP: Anteroposterior
BMI: Body mass index
CNN: Convolutional neural network
CT: Computed tomography
FMA: Femur mechanical axis
HKA: Hip knee alignment
IRB: Institutional review board
ML: Mediolateral
PTS: Posterior tibial slope
TKA: Total knee arthroplasty
TMA: Tibia mechanical axis
WHO: World Health Organization

## References

[1] Klag EA, Lizzio VA, Charters MA, Ayoola AS, Wesemann L, Banka TR, North WT (2023) Increased accuracy in templating for total knee arthroplasty using 3D models generated from radiographs. J Knee Surg 36(8):837–842. 10.1055/s-0042-1743496 35240715 10.1055/s-0042-1743496

[2] Peek AC, Bloch B, Auld J (2012) How useful is templating for total knee replacement component sizing? Knee 19(4):266–269. 10.1016/j.knee.2011.03.010 21561779 10.1016/j.knee.2011.03.010

[3] Kunze KN, Polce EM, Patel A, Courtney PM, Levine BR (2021) Validation and performance of a machine-learning derived prediction guide for total knee arthroplasty component sizing. Arch Orthop Trauma Surg 141(12):2235–2244. 10.1007/s00402-021-04041-5 34255175 10.1007/s00402-021-04041-5

[4] Kanna R, Ravichandran C, Shetty GM (2021) Notching is less, if femoral component sagittal positioning is planned perpendicular to distal femur anterior cortex axis, in navigated TKA. Knee Surg Relat Res 33(1):46. 10.1186/s43019-021-00129-9 34952652 10.1186/s43019-021-00129-9 PMC8709981

[5] Culler SD, Martin GM, Swearingen A (2017) Comparison of adverse events rates and hospital cost between customized individually made implants and standard off-the-shelf implants for total knee arthroplasty. Arthroplast Today 3(4):257–263. 10.1016/j.artd.2017.05.001 29204493 10.1016/j.artd.2017.05.001 PMC5712025

[6] Hsu AR, Gross CE, Bhatia S, Levine BR (2012) Template-directed instrumentation in total knee arthroplasty: cost savings analysis. Orthopedics 35(11):e1596–e1600. 10.3928/01477447-20121023-15 23127449 10.3928/01477447-20121023-15

[7] Riechelmann F, Lettner H, Mayr R, Tandogan R, Dammerer D, Liebensteiner M (2023) Imprecise prediction of implant sizes with preoperative 2D digital templating in total knee arthroplasty. Arch Orthop Trauma Surg 143(8):4705–4711. 10.1007/s00402-023-04772-7 36648539 10.1007/s00402-023-04772-7 PMC10374828

[8] McLawhorn AS, Carroll KM, Blevins JL, DeNegre ST, Mayman DJ, Jerabek SA (2015) Template-directed instrumentation reduces cost and improves efficiency for total knee arthroplasty: an economic decision analysis and pilot study. J Arthroplasty 30(10):1699–1704. 10.1016/j.arth.2015.04.043 26021908 10.1016/j.arth.2015.04.043

[9] Marchand KB, Salem HS, Mathew KK, Harwin SF, Mont MA, Marchand RC (2022) The accuracy of computed tomography-based, three-dimensional implant planning in robotic-assisted total knee arthroplasty. J Knee Surg 35(14):1587–1594. 10.1055/s-0041-1729548 33932948 10.1055/s-0041-1729548

[10] Seaver T, McAlpine K, Garcia E, Niu R, Smith EL (2020) Algorithm based automatic templating is less accurate than manual digital templating in total knee arthroplasty. J Orthop Res 38(7):1472–1476. 10.1002/jor.24696 32293739 10.1002/jor.24696

[11] Masse V, Ghate RS (2021) Using standard X-ray images to create 3D digital bone models and patient-matched guides for aiding implant positioning and sizing in total knee arthroplasty. Comput Assist Surg 26(1):31–40. 10.1080/24699322.2021.1894239 10.1080/24699322.2021.1894239 33721547

[12] Jo C, Hwang D, Ko S, Yang MH, Lee MC, Han HS, Ro DH (2023) Deep learning-based landmark recognition and angle measurement of full-leg plain radiographs can be adopted to assess lower extremity alignment. Knee Surg Sport Traumatol Arthrosc 31(4):1388–1397. 10.1007/s00167-022-07124-x 10.1007/s00167-022-07124-x 36006418

[13] Cao H, Wang Y, Chen J, Jiang D, Zhang X, Tian Q, Wang M (2022) Swin-unet: unet-like pure transformer for medical image segmentation. In: Cao H, Wang Y, Chen J, Jiang D, Zhang X, Tian Q, Wang M (eds) European conference on computer vision. Springer, Berlin, pp 205–18

[14] Gao ZX, Long NJ, Zhang SY, Yu W, Dai YX, Xiao C (2020) Comparison of kinematic alignment and mechanical alignment in total knee arthroplasty: a meta-analysis of randomized controlled clinical trials. Orthop Surg 12(6):1567–1578. 10.1111/os.12826 33099892 10.1111/os.12826 PMC7767667

[15] Geng Z, Sun K, Xiao B, Zhang Z, Wang J. (2021) Bottom-up human pose estimation via disentangled keypoint regression. Proceedings of the IEEE/CVF conference on computer vision and pattern recognition. 14676–86.

[16] Landis JR, Koch GG (1977) The measurement of observer agreement for categorical data. Biometrics 33(1):159–174 843571

[17] Blevins JL, Rao V, Chiu YF, Lyman S, Westrich GH (2020) Predicting implant size in total knee arthroplasty using demographic variables. Bone Joint J. 10.1302/0301-620x.102b6.Bjj-2019-1620.R1 32475285 10.1302/0301-620X.102B6.BJJ-2019-1620.R1

[18] Naylor BH, Butler JT, Kuczynski B, Bohm AR, Scuderi GR (2023) Can component size in total knee arthroplasty be predicted preoperatively?-an analysis of patient characteristics. J Knee Surg 36(9):965–970. 10.1055/s-0042-1748902 35820432 10.1055/s-0042-1748902

[19] Pietrzak JRT, Rowan FE, Kayani B, Donaldson MJ, Huq SS, Haddad FS (2019) Preoperative CT-based three-dimensional templating in robot-assisted total knee arthroplasty more accurately predicts implant sizes than two-dimensional templating. J Knee Surg 32(7):642–648. 10.1055/s-0038-1666829 30068010 10.1055/s-0038-1666829

